# Influence of Oak Species, Toasting Degree, and Aging Time on the Differentiation of Brandies Using a Chemometrics Approach Based on Phenolic Compound UHPLC Fingerprints

**DOI:** 10.1021/acs.jafc.3c00501

**Published:** 2023-05-02

**Authors:** María Guerrero-Chanivet, Fidel Ortega-Gavilán, M. Gracia Bagur-González, Manuel J. Valcárcel-Muñoz, M. Valme García-Moreno, Dominico A. Guillén-Sánchez

**Affiliations:** † Department of Analytical Chemistry, Faculty of Science, IVAGRO, Campus of Puerto Real, University of Cádiz, Puerto Real, 11510 Cádiz, Spain; ‡ Bodegas Fundador S.L.U., C/San Ildefonso, n 3, Jerez de la Frontera, 11403 Cádiz, Spain; § Department of Analytical Chemistry, Faculty of Sciences, University of Granada, Ave. Fuentenueva s/n, 18071 Granada, Spain

**Keywords:** brandy, oak, aging, phenolic compounds, fingerprint, chemometrics

## Abstract

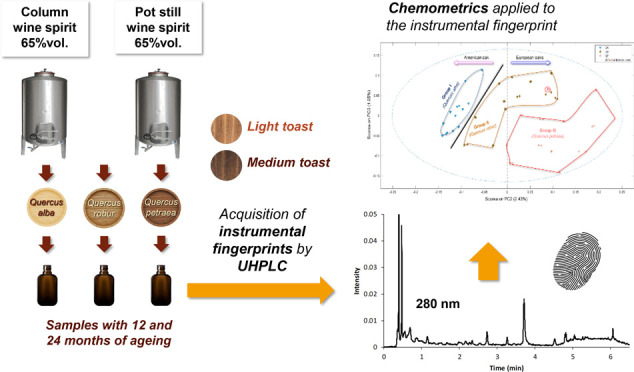

Oak wood is the main material used by coopers to manufacture casks for the aging of spirits or wines. Phenolic compounds are the main components extracted from the wood during spirit aging. In the present study, a chemometric approach based on unsupervised (PCA) and supervised (PLS–DA) pattern recognition techniques has been applied to the chromatographic instrumental fingerprints, obtained by ultra-high-performance liquid chromatography (UHPLC) at 280 nm, of the phenolic profiles of brandies aged in casks made of different oak wood species. The resulting natural data groupings and the PLS–DA models have revealed that the oak wood species, the toasting level, and the aging time are the most influential factors on the phenolic profile of the final products. Fingerprinting should be considered as a very useful feature, as it represents a considerable advantage, in terms of internal and quality control, for brandy producers.

## 1 Introduction

Brandy is a spirit with a content of at least 36% alcohol by volume (ABV). It is made from wine spirit to which wine distillate (at less than 94.8% ABV) may be added, provided that wine distillate does not exceed a maximum of 50% of the alcoholic strength of the finished brandy, and which is aged in oak wood casks for a minimum of six months when the casks are under a 1000 L volume.^1^ During the aging process, brandies gain the complexity and characteristics of old brandies. Any of the freshness or fruitiness that might have been found in the raw material vanishes, and new aromas are incorporated to this spirit, such as vanilla, smoky, toasted, or dried fruit aromas, which are closely associated with the quality of each brandy.^2,3^

Oak wood is the most commonly used material both for the manufacturing of barrels and as wood chips intended to age wines or spirits. *Quercus alba*, *Quercus robur*, and *Quercus petraea* are three of the botanical species most appreciated by cooperage companies. These species from diverse geographical origins exhibit specific wood compositions that in turn exert a particular impact on the sensory profile of the wines or spirits aged in the casks manufactured with their wood. Phenolic and furanic compounds are the main components extracted from oak wood during the aging of spirits, and their concentrations and proportions vary according to numerous factors, among which botanical species,^4−7^ toasting level,^5,8,9^ cask volume, and aging time seem to be the most relevant.

Wood is composed of 90% polysaccharides (cellulose and hemicelluloses) and lignin, while the remaining 10% consists of tannins, other phenolic compounds, short-chain carboxylic acids, fatty acids, alcohols, and inorganic substances.^10^ The phenolic compounds and furanic aldehydes that can be found in aged brandies are mainly derived from the wood that the spirit has been aged in contact with. Thus, as casks are manufactured and subjected to thermal treatments, a degradation of lignin takes place that promotes the wood contribution into the spirit with aldehydes such as vanillin, coniferaldehyde, syringaldehyde, sinapaldehyde, as well as cinnamic or benzoic acids.^9−11^ At the same time, the thermal degradation of the hemicellulose enhances the transfer of furfural and other derivatives into the drink.^12,13^ Nevertheless, furfural can also be found in certain young unaged wine spirits in varying concentrations, as they can be generated as a result of the previous distillation process that wine is subjected to.^14,15^ This will also represent a likely influence on the content of the final aged product.

American oak, *Quercus alba*, is the wood variety that is most often used for the production of Sherry Brandy in its Protected Geographic Indication (PGI). Nonetheless, other oak European species, such as *Quercus petraea* and *Quercus robur*, can also be found used for the aging of these tasty spirits. The casks employed for this purpose are generally made of medium toast staves, but some casks more intensely toasted can also be identified. The level of toasting is a crucial factor with regard to the transferring of different types of compounds into the aged products.

Other production stages and/or factors also play an important role with respect to the chemical-sensory profile of the resulting brandies. The distillation method employed to produce the young wine spirit particularly stands out among them.^3,16,17^ With this regard, two are the most commonly used distillation techniques, namely, continuous column^3,15,18^ or pot still distillation either in one or two steps.^17^ Together with the aging process itself, the method used to produce the unaged wine spirit is one of the most important steps in the whole brandy production process, because of its major impact on the profile of the aged product.

The agrifood sector is applying fingerprinting to a growing number of applications. This novel strategy consists in combining instrumental fingerprinting with chemometric techniques.^19−22^ According to the conditions under which the samples are analyzed, instrumental fingerprints allow the association of its results to a single and specific sample category. Since the composition of the samples is determined in a nonselective way, it is unnecessary to identify or quantify each of the compounds that are present in the sample. Consequently, through the chemometric study of the instrumental fingerprints of the brandies analyzed, not only can we evaluate the natural groupings that take place, but classification models can also be developed to be used as regular quality control methods in wineries.

In this study, ultra-high-performance liquid chromatography (UHPLC) was used to acquire the instrumental fingerprints of the phenolic and furfural profiles at 280 nm of aged brandy samples. The chromatographic fingerprints of more than 70 samples of brandies produced from two different distillates, aged for 12 or 24 months in 350 L casks made of wood from three different oak species (*Quercus alba*, *Quercus robur*, or *Quercus petraea*) previously toasted at two different levels (medium or light), have been recorded and preprocessed under a chemometric approach focused on pattern recognition.

## 2 Material and Methods

### 2.1 Samples

The wine spirits used for this study were obtained from wines of the Airén grape variety (Castilla La Mancha, Spain) all of which were suitable for distillation. Two distillation methods have been employed: continuous column distillation and two pot stills in series. This resulted in two types of wine spirits that complied with the technical specifications set out in the governing regulations.^1^ The brandy obtained by column distillation reached 77% ABV, while the one obtained through the pot stills contained 65% ABV. Both wine spirits were adjusted to 65% ABV before their aging. For this purpose, the wine spirit that had been obtained by continuous column distillation was hydrated using demineralized water until the desired alcoholic strength was obtained.

The 350 L casks used to age the brandies were made of three different oak wood species: *Quercus alba*, *Quercus robur*, and *Quercus petraea*. The wood for these casks had undergone medium and light toasting treatments. In order to assess the evolution of the brandies, they were sampled after 12 and 24 months of aging. A total of 72 cases have been studied. All of the samples taken have been analyzed in duplicate.

The wine spirits, the oak casks, and the facilities where the experiments were carried out were provided by the company Bodegas Fundador, S.L.U.

### 2.2 Chemicals and Reagents

The standards used for the identification of some of the compounds present in the analyzed fraction were gallic acid (certified reference material); 5-hydroxymethylfurfural (≥99%); furfural (≥98.5%, GC); vanillic acid (≥97.0%, HPLC); *p*-hydroxybenzaldehyde (95.0%, HPLC); 5-hydroxymethylfurfural (≥99%); syringic acid (≥95.0%, HPLC); vanillin (≥97%); syringaldehyde (≥98%); coniferaldehyde (≥98%); and sinapaldehyde (≥98%), all of which were supplied by Sigma-Aldrich (Saint Louis, MO).

The hydroalcoholic mixtures used for the identifications were made using 99.8% ethanol supplied by Sigma-Aldrich (Saint Louis, MO) and ultrapure water (EMD-Milipore, Bedford, MA).

### 2.3 UHPLC Analysis

A Waters Acquity UPLC system fitted with a PDA detector was used for the chromatographic analyses. The stationary phase was a 100 × 2.1 mm (i.d.) with 1.7 μm particle size Acquity UPLC C18 BEH column (Waters Corporation, Milford, MA). The chromatographic conditions were selected according to those proposed by Schwarz et al.^23^ Finally, the chromatograms were extracted at 280 nm wavelength.

### 2.4 Data Processing

The data were acquired by the software application Empower 3 (Waters Corporation, Milford, MA). In order to generate the chromatographic profiles, i.e., the instrumental fingerprints, all of the chromatograms extracted at 280 nm were exported into CSV format files. For the construction of the brandies’ fingerprint matrices, the procedure described by Bagur-González et al.^19^ was followed.

Two 72 × 7799 fingerprint matrices were obtained for the phenolic and the furfural compounds, separately. The data were preprocessed by means of MATLAB, R2013b version (Mathworks Inc., Natick, MA) by applying the ad hoc script known as Medina (version 14)^24^ in accordance with the procedure described in previous works by our research team.^20,22^ The Medina script provides access to a number of functions in MATLAB Bioinformatics Toolbox that allow filtering, smoothening, or correcting the signal baseline. As a final step, the script makes use of an “icoshift” algorithm to align the peaks in the chromatograms.^25^

Before the pattern recognition techniques were applied, each matrix was mean centered by means of PLS_Toolbox, as a final preprocessing stage.

PLS_Toolbox was also used to conduct the principal component analysis (PCA) and the partial least squares–discriminant analysis (PLS–DA).

## 3 Results and Discussion

In order to determine the degree of influence of the oak wood type on the phenolic composition of the aged brandies, two types of wine spirits obtained through different distillation methods (column and pot still) and three wood types (*Quercus alba*, *Quercus robur*, and *Quercus petraea*), subjected to two levels of toasting (medium and light), were used in this study. In all cases, in order to evaluate the evolution of the instrumental fingerprints, two aging times were selected, namely, 12 and 24 months. All of these variables were taken into account throughout the different pattern recognition examinations.

**Figure 1:**
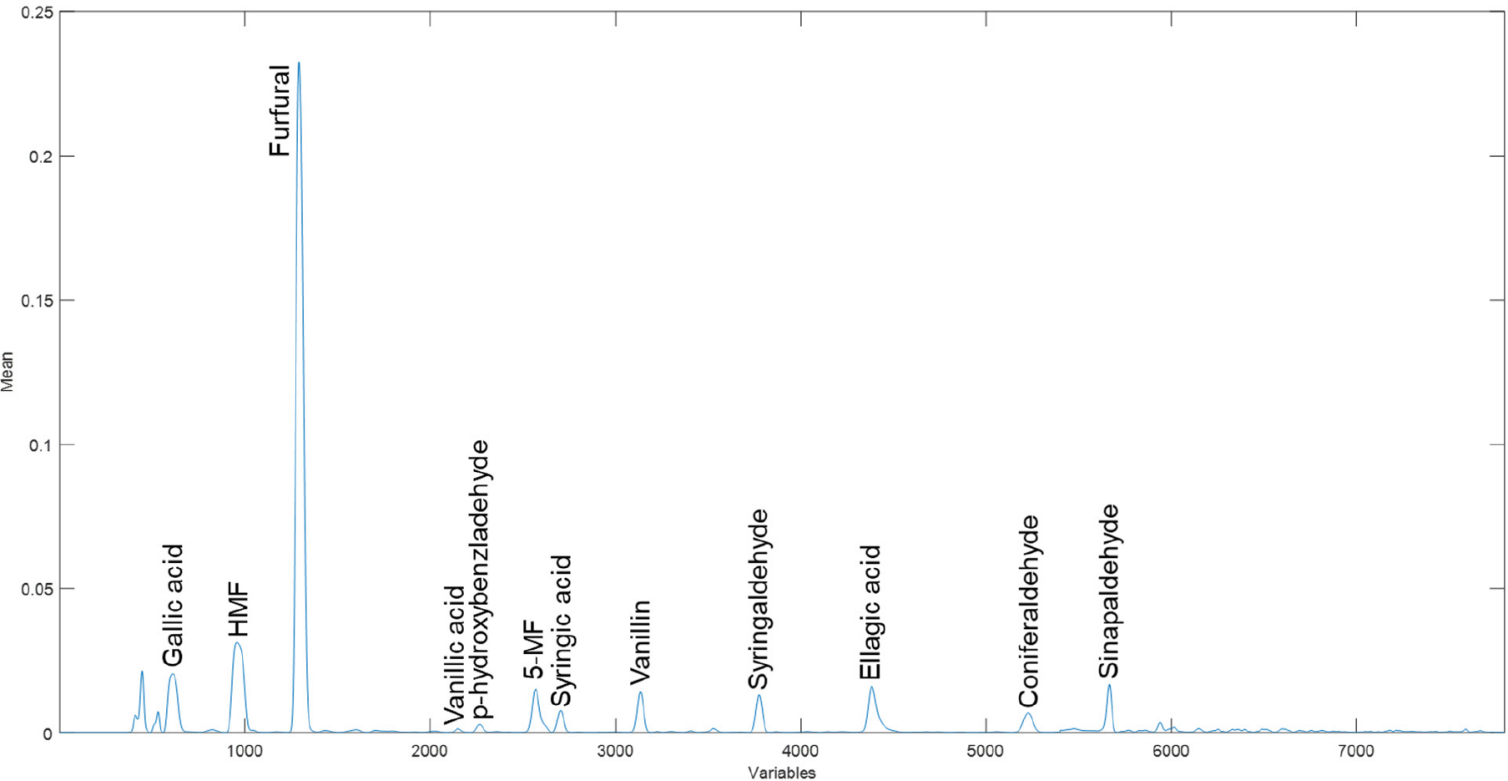
Average instrumental fingerprint of phenolic compounds of the brandies analyzed. HMF, 5-hydroxymethyl furfural; 5-MF, 5-methylfurfural.

A representative fingerprint of the compounds that were analyzed can be seen in Figure 1, where the known chromatogram peaks have been indicated.

### 3.1 Unsupervised Pattern Recognition Analysis: Principal Component Analysis (PCA)

When PCA was applied to the matrix of the instrumental fingerprints concerning the phenolic and furfural compounds in the brandies, 3 principal components (PCs) were selected that explained 99.48% of the total variance of the model. The projection on PC1 explains 96.00% of the total variance of the model, while the other two components explain 2.43% (PC2) and 1.05%, respectively, of the remaining variance (PC3).

**Figure 2:**
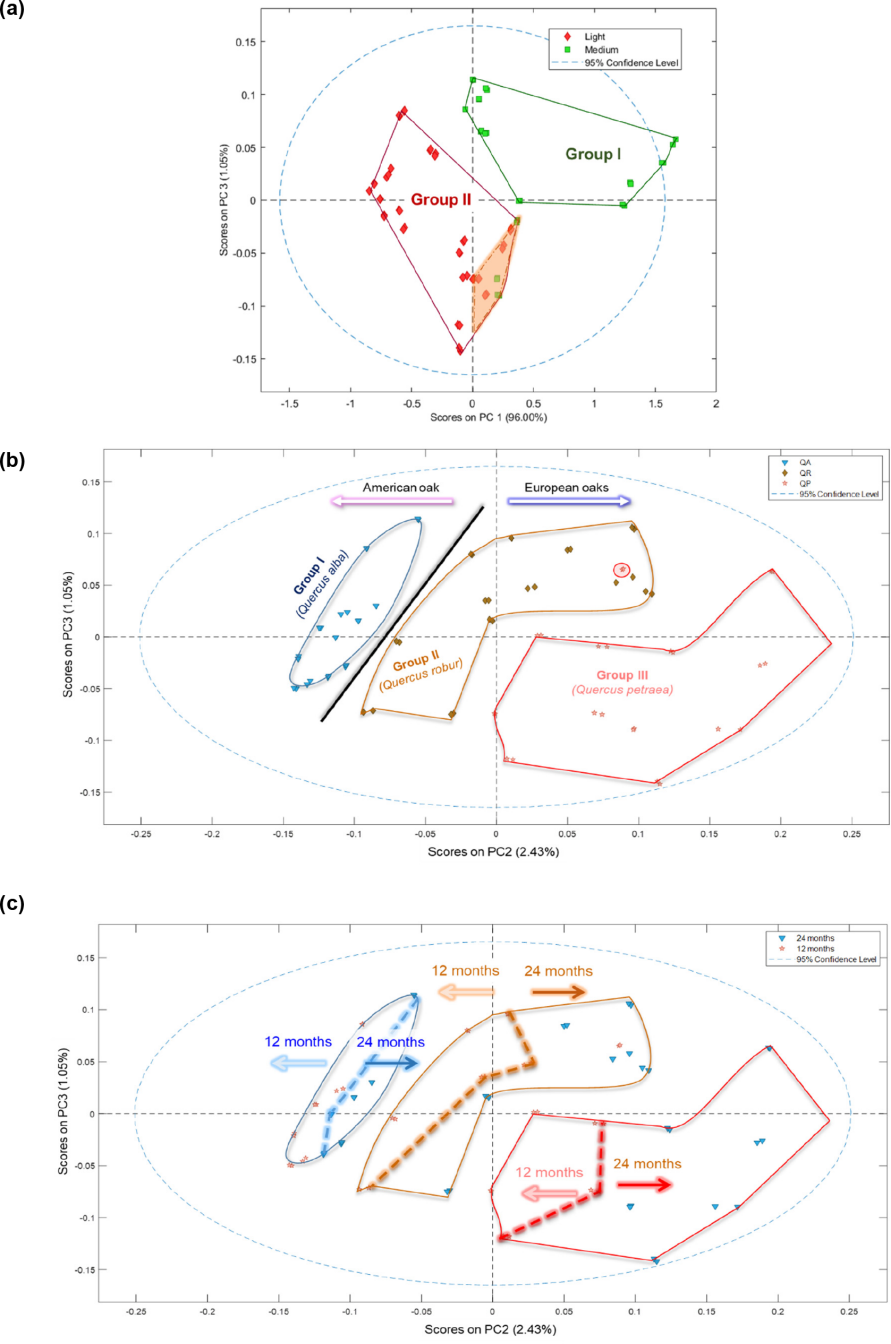
Score plots of the brandies in (a) the plane PC3 vs PC1 selecting the roasting level as the relevant attribute, (b) the plane PC3 vs PC2 selecting the oak species as the relevant attribute, and (c) the plane PC3 vs PC2 selecting the aging time as the relevant attribute.

Figure 2a–c displays the scores given to the brandies through different graphical projections corresponding to the space of the three components selected on the basis of specific attributes.

In Figure 2a (PC3 vs PC1), where the type of toast of the casks’ wood is considered the factor of interest, two large groups can be observed. The first of these (Group I) is constituted mainly by those casks that had been subjected to a medium toast. They are characterized by their positive or close to zero scores for both PCs. The second (Group II), with mostly negative scores for PC1 and positive or negative scores for PC3, consisted mainly of those brandy samples aged in the oak casks that had been given a light toasting treatment. Since the toasting process is entirely manual, it should be noted that the variability between light toast treatments is much more noticeable than that corresponding to the medium toast treatments. Thus, these casks appear scattered around three of the four quadrants of the new projection space. Actually, the samples included in the second quadrant (area shaded in pale orange inside Group II) are those where the type of toasting treatment received is not so clearly distinguished.

Figure 2b shows a trend to group the samples into two clusters. These clusters match the geographical origin of the oak wood used to age the brandies, i.e., American (*Quercus alba*) or European (*Quercus robur* or *Quercus petraea*) oak woods. Such grouping may be explained both by the similarity between these two European species, and their similar phenolic profiles, and by the proximity of their geographical origin, since both species come mainly from the south of France and north of Spain.^5^ In addition, three groups that correspond to the botanical origin of the oak variety also appear: Group I is formed by the samples aged in casks made of *Quercus alba* wood. Group II includes those samples aged in casks made of *Quercus robur* wood. Finally, Group III is constituted by the samples aged in a cask of the *Quercus petraea* species.

Finally, Figure 2c shows how within each of the groups described above, when the aging time of the brandies corresponding to each wood type is the factor to be considered, it can be observed that the samples within each group present a tendency to regroup. Thus, the brandies aged for 12 months appear in the left area within each of the groups.

**Figure 3:**
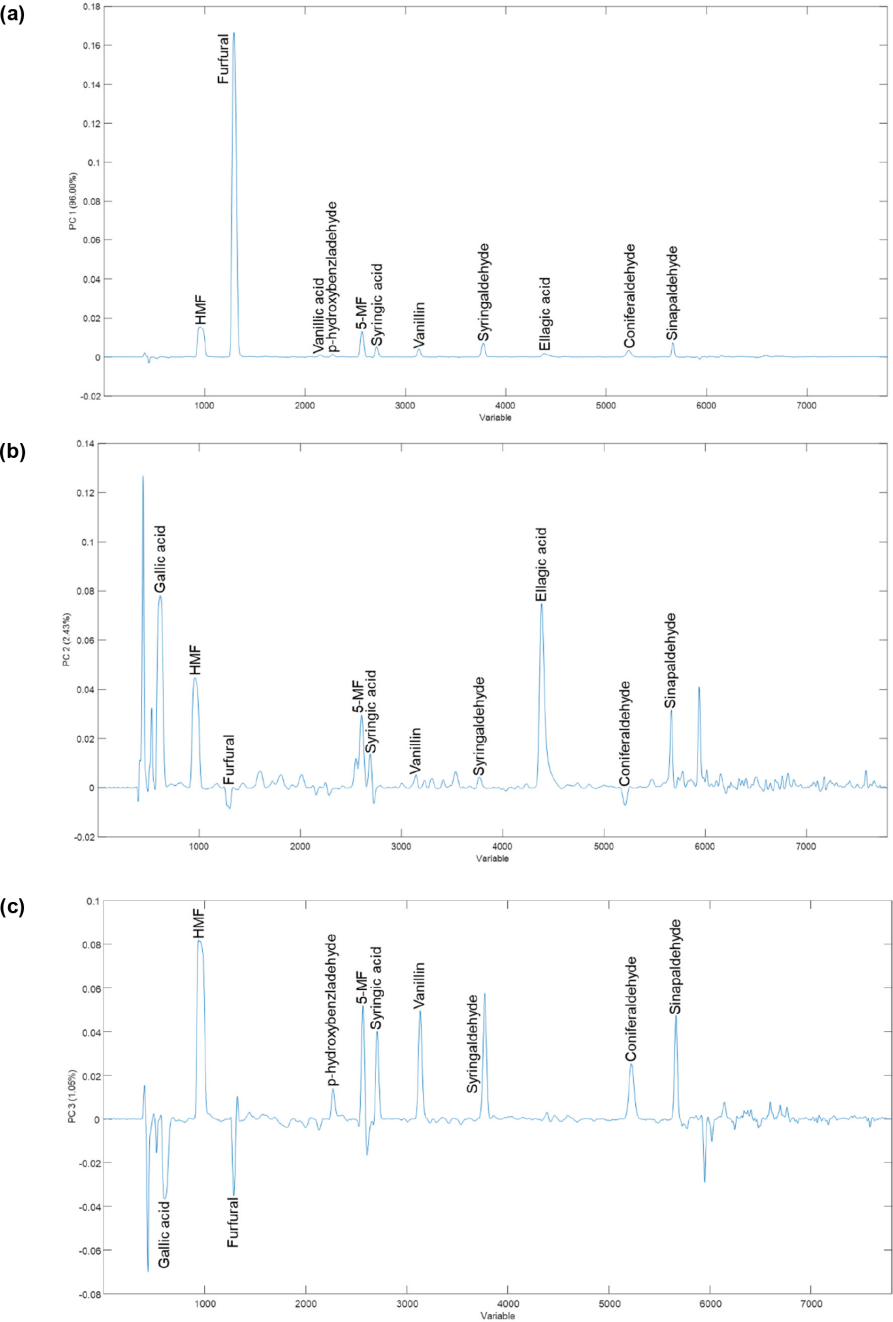
Loading plots for (a) PC1, (b) PC2, and (c) PC3.

Figure 3a–c shows the loading plots for the PCs that characterize the clustering model. These figures show the following:

(i) With respect to PC1, the areas of the fingerprint that exhibit the greatest influences on the clusters found are associated with furfural and its derivatives (HMF and 5-MF). Given that these compounds come from the degradation of the hemicellulose in the wood,^12,13^ they are greatly affected by the toasting treatment of the barrel and are even closely related to both the unaged distillate and the distillation method.^14,15^ This component is dependent to a lesser extent on the other zones associated with guaiacyl-type aldehydes (vanillin and coniferylaldehyde) or syringyl-type aldehydes (syringaldehyde and sinapaldehyde). These come from the degradation of another major component in wood, lignin,^9,10^ and are also closely related to the toasting level of the casks, so that it can be found mainly in the brandies that had been aged in medium toasted casks.
(ii) Regarding PC2 (Figure 3b), this is a component that allows us to distinguish the wood type and the aging time of the brandies analyzed. It should also be noted that the areas of the fingerprint that exhibit the greatest influence are those where gallic acid, HMF, ellagic acid, 5-MF, syringic acid, and sinapaldehyde appear. This fact is in agreement with several reports by other authors^6,7^ who associate a substantial variability in the phenolic composition of the final brandies to the botanical origin of the aging wood. Thus, *Quercus alba* wood is less rich in hydrolyzable tannins and, therefore, in gallic and ellagic acid than the varieties *Quercus robur* or *Quercus petraea*, while the American species is richer in vanillin-type aldehydes. According to the authors’ opinion, this would explain the fact that this component, despite its minor contribution to the total variance of the model, allows the differentiation between oak varieties. On the other hand, it is well-known that American oak has smaller pore openings than either of the European species (*Quercus robur* and *Quercus petraea*). American oak is denser and less porous than European oak; it also contains a higher content of tylose lignin, an effective coagulating agent that clogs pores.^28,31^ This fact makes the extraction of wood compounds from it more difficult. This fact would explain why the brandies aged in American oak are easier to differentiate from the other brandies aged in oak wood from European origin.
(iii) In relation to the PC3 loadings (Figure 3c), it should be noted that the groupings found when the attributes oak type and aging time were considered were explained by significant variations in the gallic acid and furfural acid contents of the brandies studied, in addition to the above.

### 3.2 Supervised Pattern Recognition Analysis: Partial Least Squares–Discriminant Analysis (PLS–DA)

In order to verify that the clusters obtained through the unsupervised pattern recognition analysis allow regarding the experimental variables studied as classificatory, different binary (one input class) discrimination models were developed through PLS–DA.

In all the cases, the original instrumental fingerprint matrices were divided into two subsets: (i) the first one, the training set, consisting in a matrix of 51 instrumental fingerprints was used to establish the model and for internal cross-validation (Venetian blinds, data Split 10), and (ii) the second subset (the external validation set) was made up of a matrix of 21 instrumental fingerprints intended for the external validation of the models in the prediction stage. In all the cases, the samples were distributed according to the Kennard–Stone algorithm. The instrumental fingerprints of the phenolic and furfural compounds, which are affected by the variables (i) wood toast level, (ii) oak wood type, and (iii) aging time, were used to construct all of the models below.

#### 3.2.1 PLS–DA Model According to the Toasting Level of the Brandies’ Aging Casks

This model was constructed using the matrix of the instrumental fingerprints corresponding to the phenolic and furfural compounds, where the medium toasting of the aging casks was considered as an input class. Ten latent variables were selected that explained 99.91% of the total variance in the matrix of the instrumental fingerprints that had been used for the training stage and 95.38% of the total variance of the class.

**Figure 4:**
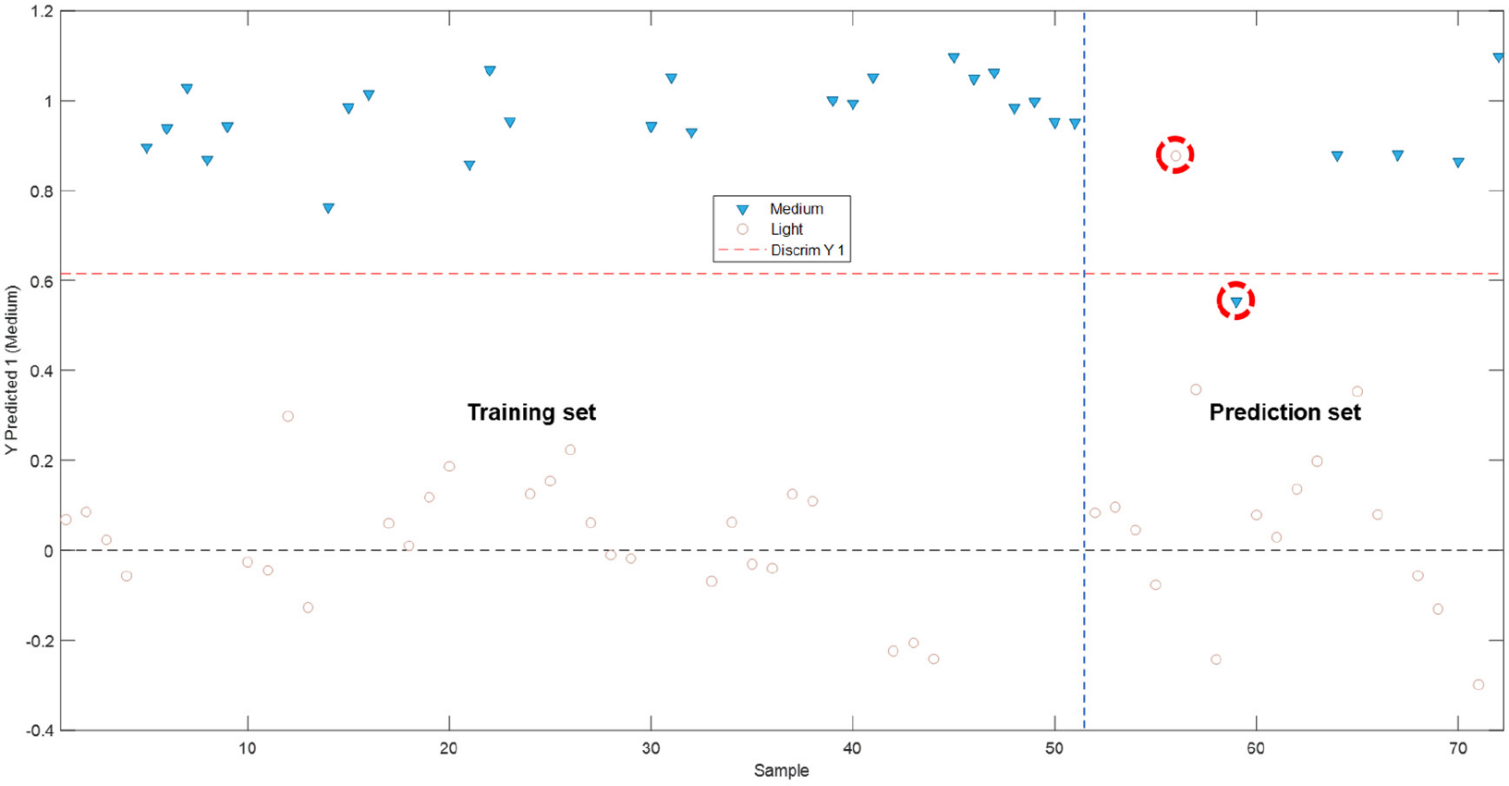
Binary classification plots obtained from the PLS–DA model light/medium toast.

When the classification graph (Figure 4) was examined, it could be seen that the model established allowed the instrumental fingerprint of the phenolic and furfural compounds to be used to successfully discriminate/classify and predict brandies aged in casks with a medium toast. In fact, only two of the samples used in the prediction set (marked in the figure by a red dotted circle) were misclassified, and they had no specific relationship between them. As already mentioned in the previous sections, cask toasting is an artisanal practice that leads to a certain heterogeneity between the processed products. This can contribute to the misclassification of certain samples. This behavior suggests that, when a cask is subjected to a medium toasting process, the varying final level of toasting that is obtained may, in some cases, come closer to a light toasting treatment rather than to a medium one, and vice versa. On the other hand, given the quality metrics of the proposed model (Table 1), such a model would allow the determination of the level of toasting of the casks used in a winery.

**Table 1:** Summary of Discrimination/Classification Performance Metrics Obtained for the Fourth PLS–DA Binary Model

Features
*X Block: [Phenolic and furfural instrumental fingerprints]
*Y Block: [TC (medium); NTC (not medium; i.e., light)]
Preprocessing: Mean center
Training Set: [51 × 7799]
Prediction Set: [21 × 7799]

Classification performance metrics	TC^a^ (medium)	NTC^b^ (not medium)
*R*^2^ (calibration stage)	0.95	0.95
*R*^2^ (cross-validation stage)	0.77	0.77
*R*^2^ (prediction/external validation stage)	0.63	0.63
RMSE (calibration stage)	0.11	0.11
RMSE (cross-validation stage)	0.25	0.25
RMSE (prediction/external validation stage)	0.27	0.27
Sensitivity (SENS -prediction stage)	0.92	1.00
Specificity (SPEC-prediction stage)	1.00	0.92

a TC: target class.

b NTC: not target class.

#### 3.2.2 PLS–DA Model According to the Brandy Aging Casks Wood Types

**Figure 5:**
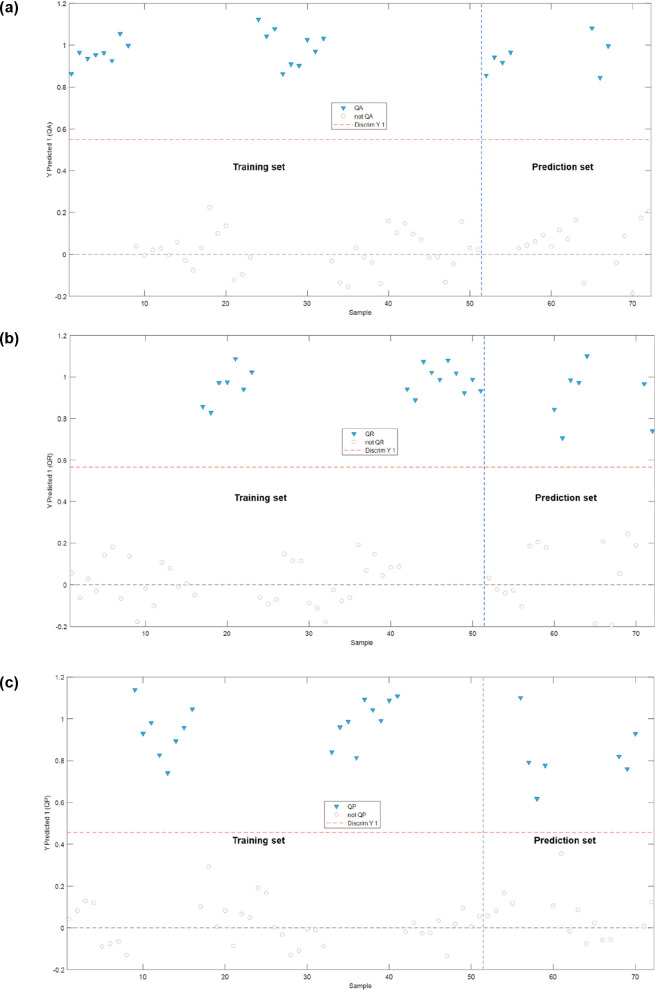
Binary classification plots obtained from the PLS–DA models: (a) QA–non-QA; (b) QR–non-QR; and (c) QP–non-QP. QA, *Quercus alba*; QR, *Quercus robur*; QP, *Quercus petraea*.

Three binary models (one input class) were generated in order to discriminate between the brandies aged in the different oak woods, according to the following categories: QA (*Quercus alba*), QR (*Quercus robur*), and QP (*Quercus petraea*). The binary classification plots obtained for the three models are presented in Figure 5a–c.

The model intended to discriminate between the brandies aged in American oak, *Quercus alba*, or either of the European oaks, *Quercus robur* or *Quercus petraea* (QA vs non-QA), was constructed based on 7 latent variables that explained 99.81% of the variance of the instrumental fingerprints of the samples and 96.46% of the variance of the modeled class. When looking into the binary classification plot of this model (Figure 5a), it can be observed that both sample sets, the one used for the training as well as the one used for prediction, appear correctly assigned to the modeled class.

The model intended to discriminate between the brandies aged in *Quercus robur* versus those aged either in *Quercus alba* or *Quercus petraea* (QR vs non-QR) was constructed using 10 latent variables that explained 99.89% of the variance of the instrumental fingerprints of the samples and 95.98% of the variance of the modeled class. From the binary classification plot of this model (Figure 5b), it could be seen that both sets of samples, the ones used for the training as well as those used for prediction, appeared correctly assigned to the modeled class.

Finally, Figure 5c displays the binary classification plot of the model constructed by taking *Quercus petraea* as the input class. This model was constructed by selecting 8 latent variables that explained 99.84% of the total variance of the samples and 95.08% of the total variance of the class. As with the two other previous models, it can be considered that the model discriminates/classifies correctly, since the cross-validation and prediction samples were not misclassified.

These three models corroborate once again that the variation in the chromatographic fingerprint of the phenolic and furfural compounds allows a clear discrimination between the brandies aged in each of the three oak wood types used: *Quercus alba*, *Quercus robur*, or *Quercus petraea*.

The quality metrics of the different models developed are included in Tables 2a–2c.

**Table 2a:** Summary of Discrimination/Classification Performance Metrics Obtained for the First PLS–DA Binary Model

Features
*X Block: [Phenolic and furfural instrumental fingerprints]
*Y Block: [TC (QA); NTC (non-QA; i.e., QR and QP)]
Preprocessing: Mean center
Training Set: [51 × 7799]
Prediction Set: [21 × 7799]

Classification performance metrics	TC^a^ (QA)	NTC^b^ (non-QA)
*R*^2^ (calibration stage)	0.96	0.96
*R*^2^ (cross-validation stage)	0.88	0.88
*R*^2^ (prediction/external validation stage)	0.95	0.95
RMSE (calibration stage)	0.09	0.09
RMSE (cross-validation stage)	0.16	0.16
RMSE (prediction/external validation stage)	0.11	0.11
Sensitivity (SENS -prediction stage)	1.00	1.00
Specificity (SPEC-prediction stage)	1.00	1.00

a TC: target class.

b NTC: not target class.

**Table 2b:** Summary of Discrimination/Classification Performance Metrics Obtained for the Second PLS–DA Binary Model

Features
*X Block: [Phenolic and furfural instrumental fingerprints]
*Y Block: [TC (QR); NTC (non-QR; i.e., QA and QP)]
Preprocessing: Mean center
Training Set: [51 × 7799]
Prediction Set: [21 × 7799]

Classification performance metrics	TC^a^ (QR)	NTC^b^ (non-QR)
*R*^2^ (calibration stage)	0.96	0.96
*R*^2^ (cross-validation stage)	0.72	0.72
*R*^2^ (prediction/external validation stage)	0.89	0.89
RMSE (calibration stage)	0.09	0.09
RMSE (cross-validation stage)	0.25	0.25
RMSE (prediction/external validation stage)	0.16	0.16
Sensitivity (SENS -prediction stage)	0.94	1.00
Specificity (SPEC-prediction stage)	1.00	0.94

a TC: target class.

b NTC: not target class.

**Table 2c:** Summary of discrimination/classification performance metrics obtained for the third PLS–DA binary model

Features
*X Block: [Phenolic and furfural instrumental fingerprints]
*Y Block: [TC (QP); NTC (non-QP; i.e., QA and QR)]
Preprocessing: Mean center
Training Set: [51 × 7799]
Prediction Set: [21 × 7799]

Classification performance metrics	TC^a^ (QP)	NTC^b^ (non-QP)
*R*^2^ (calibration stage)	0.95	0.95
*R*^2^ (cross-validation stage)	0.79	0.79
*R*^2^ (prediction/external validation stage)	0.90	0.90
RMSE (calibration stage)	0.10	0.10
RMSE (cross-validation stage)	0.22	0.22
RMSE (prediction/external validation stage)	0.16	0.16
Sensitivity (SENS -prediction stage)	0.95	0.95
Specificity (SPEC-prediction stage)	0.79	0.79

a TC: target class.

b NTC: not target class.

#### 3.2.3 PLS–DA According to the Brandies’ Aging Times

Finally, we proceeded to construct a discrimination model based on the matrix of the instrumental fingerprints corresponding to the phenolic and furfural compounds, considering 12 month aging as the input class, since aging time is one of the parameters that affect the phenolic content in brandies. For this purpose, 8 latent variables that explained 99.87% of the total variance in the matrix of the instrumental fingerprints used to train the model and 95.42% of the total variance of the class were employed.

**Figure 6:**
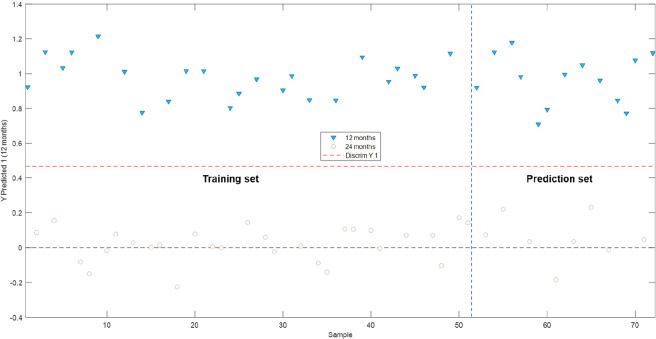
Binary classification plots obtained from the PLS–DA model after 12 months/24 months of aging.

When the classification graph (Figure 6) was examined, it could again be observed that the established model allowed the use of the instrumental fingerprints corresponding to the phenolic and furfural compounds in the brandies to correctly discriminate and predict their aging time. Since brandy is enriched by the extraction or release of these compounds from the wood, the longer the aging time, the greater the brandy is enriched in phenolic and furfural compounds. This is the reason why the model that had been developed proved to be a reliable method to accurately discriminate between the set of samples that had been aged for 12 months and the one that comprised 24 month old brandy samples.

The quality metrics of the proposed model are included in Table 3.

**Table 3:** Summary of Discrimination/Classification Performance Metrics Obtained for the Fifth PLS–DA Binary Model

Features
*X Block: [Phenolic and furfural instrumental fingerprints]
*Y Block: [TC (12 months); NTC (not 12 months; i.e., 24 months)]
Preprocessing: Mean center
Training Set: [51 × 7799]
Prediction Set: [21 × 7799]

Classification performance metrics	TC^a^ (12 months)	NTC^b^ (not 12 months)
*R*^2^ (calibration stage)	0.95	0.95
*R*^2^ (cross-validation stage)	0.86	0.86
*R*^2^ (prediction/external validation stage)	0.92	0.92
RMSE (calibration stage)	0.11	0.11
RMSE (cross-validation stage)	0.19	0.19
RMSE (prediction/external validation stage)	0.14	0.14
Sensitivity (SENS -prediction stage)	1.00	1.00
Specificity (SPEC-prediction stage)	1.00	1.00

a TC: target class.

b NTC: not target class.

In summary, the unsupervised pattern recognition technique that had been applied (PCA) allowed the observation of the natural groupings that took place based on toast level, oak species, and aging time, with the first two variables having the greatest impact on the natural grouping of the brandies. Regarding the supervised chemometric analysis by means of PLS–DA, the discrimination between brandies according to oak species, toast level, and aging time was also successfully achieved. This study has confirmed the impact of the aforementioned variables on the instrumental fingerprints of the phenolic and furfural compounds that are found in brandies. It should also be noted that fingerprinting has proven to be highly reliable for the analysis of this kind of matrix, as it allows taking into account not only known compounds but also unidentified compounds that may appear in specific areas and that are associated with the fingerprints of the brandies under study. This should be considered as a very useful feature, as it represents a considerable advantage, in terms of internal control, for brandy producers.
